# PET/MRI in Oncological Imaging: State of the Art

**DOI:** 10.3390/diagnostics5030333

**Published:** 2015-07-21

**Authors:** Usman Bashir, Andrew Mallia, James Stirling, John Joemon, Jane MacKewn, Geoff Charles-Edwards, Vicky Goh, Gary J. Cook

**Affiliations:** 1 Cancer Imaging Department, Division of Imaging Sciences and Biomedical Engineering, King’s College London, London, SE1 7EH, UK; E-Mails: usman.bashir@kcl.ac.uk (U.B.); andrew.mallia@kcl.ac.uk (A.M.); james.stirling@nhs.net (J.S.); vicky.goh@kcl.ac.uk (V.G); 2 PET Imaging Centre and the Division of Imaging Sciences and Biomedical Engineering, King’s College London, London, SE1 7EH, UK; E-Mails: joemon.john@gstt.nhs.uk (J.J.); jane.mackewn@kcl.ac.uk (J.M.); 3 Medical Physics, Guy’s & St Thomas’ Hospitals NHS Foundation Trust, London, SE1 7EH, UK; E-Mail: geoff.charles-edwards@kcl.ac.uk; 4 Department of Radiology, Guy’s & St Thomas’ Hospitals NHS Foundation Trust, London, SE1 7EH, UK

**Keywords:** PET/MRI, MR-PET, cancer, diagnosis, imaging

## Abstract

Positron emission tomography (PET) combined with magnetic resonance imaging (MRI) is a hybrid technology which has recently gained interest as a potential cancer imaging tool. Compared with CT, MRI is advantageous due to its lack of ionizing radiation, superior soft-tissue contrast resolution, and wider range of acquisition sequences. Several studies have shown PET/MRI to be equivalent to PET/CT in most oncological applications, possibly superior in certain body parts, e.g., head and neck, pelvis, and in certain situations, e.g., cancer recurrence. This review will update the readers on recent advances in PET/MRI technology and review key literature, while highlighting the strengths and weaknesses of PET/MRI in cancer imaging.

## 1. Introduction

Combined positron emission tomography with magnetic resonance imaging (PET/MRI) is a promising new modality which may replace PET/CT in selected cancer scenarios and may generate new oncological applications. In PET/CT, the limited spatial resolution of PET is compensated by CT, providing valuable anatomic and morphological information complementary to the metabolic and molecular information provided by PET, making it a mainstay investigation in staging and re-staging of a wide range of cancers [1]. Some of the issues in PET/CT include the added radiation dose from CT and the limited soft-tissue contrast resolution of CT. MRI, however, not only offers superior contrast resolution between different types of soft tissues, it allows physiological (e.g., dynamic contrast enhanced MRI), metabolic (e.g., MR spectroscopy), and molecular (e.g., diffusion weighted imaging) phenomena to be observed. Based on these advantages, it would be expected that hybrid PET/MRI scanners may provide a superior solution to PET/CT in some cancer imaging applications.

Although PET/MRI has been investigational since before PET/CT, several technological problems have prevented its clinical deployment [2,3,4]. First, physically combining the two scanner types has been a challenge for a number of reasons. For example: (a) conventional photomultiplier tubes used in standard PET detectors are highly sensitive to even relatively small magnetic fields and therefore cannot be located in or even near to an MRI scanner; (b) Radiofrequency (RF) cross talk can occur between the two scanners in the absence of appropriate RF shielding; and (c) there is only a limited amount of available space inside an MR scanner in which a PET ring can be located, when considering a fully integrated system. Second, attenuation correction, which is an essential part of PET imaging and is performed using the CT data on conventional PET/CT platforms, is more complicated using an MRI image as the image intensity bears no direct relationship to tissue attenuation. Methods instead rely on tissue segmentation, *i.e.*, deriving different classes of tissue type which are then assigned to a particular attenuation factor, or Atlas based approaches.

Recent advances in engineering have addressed these issues to a large extent, and studies are underway to assess the effectiveness of PET/MRI, compared with PET/CT. Most studies indicate that cancer staging with PET/MRI is feasible and as accurate as PET/CT in most body regions; in head, neck, and pelvis, there is evidence that PET/MRI may be superior to PET/CT. A second question which is being pursued is the logistical advantage in combining PET and MRI into a single examination in situations when both are commonly performed, for example, in relapse of pelvic malignancies. In such situations there may be advantages in patient-experience, reduced radiation and cost-saving. Therefore, the purpose of this review is to:

- Provide an overview of the technical aspects of PET/MRI design.
- Describe the limitations of simultaneous PET/MRI imaging.
- Review current literature investigating the diagnostic accuracy of PET/MRI in cancer imaging compared with the conventional imaging tools, in particular, PET/CT. By order of preference, recent studies utilizing hybrid PET/MRI scanners will be mentioned before studies performed on fused PET and MRI data acquired separately.

## 2. Technical Aspects of PET/MRI

Although first experiments on PET/MRI started earlier than PET/CT, in the mid-1990s [5], clinical implementation has lagged behind PET/CT due to the greater number of technological challenges that have had to be addressed. Various PET/MRI designs have been investigated over recent years that address the issues outlined in Section 1 above. Second, attenuation correction, an essential part of any PET platform, is not as robust with PET/MRI as it is with PET/CT [6]. The following paragraphs will discuss both technical aspects of PET/MRI imaging in turn:

### 2.1. PET/MRI Design Considerations

Besides software-fusion of separately acquired MRI and PET images, there are two popular PET/MRI hardware designs, performing both studies either sequentially or simultaneously.

### 2.2. Sequential

A sequential design involves imaging on separate PET and MRI systems. The PET and MRI units may be in the same room (e.g., Phillips Ingenuity TF PET/MR) or in separate rooms (e.g., GE trimodality PET/CT/MRI). The patient remains on a mobile scanning couch and is transferred from one scanner to the other and, therefore, spatially co-registered images are acquired without the need to reposition the patient.

A sequential PET and MRI setup is the technologically least challenging and economical solution, albeit with its limitations, *i.e.*, greater space requirements to house two separate scanners (typically 4.3 × 13 m), increased time required for two separate acquisitions, and propensity for artefact as the patient may move between acquisitions [7].

### 2.3. Simultaneous

Scanners which perform PET and MRI simultaneously have been in use since 2006 [8,9,10]. However, combining PET and MRI detectors into a single gantry is, technically, the most challenging design requiring all issues described in Section I to be fully addressed. Designs of such systems include placing the PMTs at a distance from the magnetic field via long optic fibers in preclinical scanners [11], using an MR magnet with a gap housing PET detectors (“split design concept”), and more recently, using field insensitive solid-state detectors—the avalanche photo-diodes (APD)—in place of PMTs. PET/MRI scanners utilizing APD detectors in a fully-integrated PET/MRI assembly have shown promising diagnostic quality. However, these scanners do not allow time-of-flight (TOF) PET due to the low timing resolution of APD detectors. More recently, PET/MR models utilizing silicon photomultiplier tubes (SiPMT) in place of APDs have become available. These scanners are TOF-enabled because of the high timing resolution of SiPMTs.

Interested readers are pointed to the following reviews for further technological and historical details on the development of PET/MR [7,12].

### 2.4. Attenuation Correction in PET/MRI

In PET imaging, the radionuclide decays and results in the emission of two annihilation photons of 511 keV in opposite directions, along a “line of response” (LOR). Opposing PET detectors are linked to register co-incident counts as a single event. However, one or both photons from a single event may be attenuated by body tissues before being detected, causing loss of signal proportional to the depth of the annihilation event from surface and regional tissue density. Attenuation correction (AC) is a post-processing step which accounts for these attenuation variations and is essential in providing PET images of diagnostic quality and quantitative accuracy.

The present solution of CT-based transmission systems as used in PET/CT attenuation correction is not possible in PET/MRI. This is because CT measures photon attenuation and, being similar in principle to PET, allows attenuation correction of the higher energy (511 keV) gamma photons. MRI, conversely, measures signals based on proton density and is unable to provide an analogous AC to CT. Several “work-around” solutions have been devised to allow MRI-based AC, and can be broadly classified into software based AC algorithms and dedicated AC sequences. For the sake of simplification, a generalized overview of the various approaches is provided below; interested readers are referred elsewhere[13].

*Software-based algorithms*: These approaches derive AC maps for patient datasets either through the use of template data-sets or artificial intelligence algorithms. Techniques relying on templates utilize pre-available templates of normal MRI and co-registered CT-derived attenuation maps. A patient MRI scan is matched with the template MRI to generate a patient-specific mathematical transformation. This transformation is then applied to the template attenuation map to derive a patient-specific attenuation map. The utility of template-based AC is limited to brain imaging. Artificial intelligence (AI) techniques, however, do not require templates, and process patient-images to segment anatomical structures such as brain, sinuses, and bone with the help of AI algorithms (e.g., fuzzy logic and neural networks). AI techniques can be utilized in whole body imaging and are considered superior to template-based approaches.

**Figure 1:**
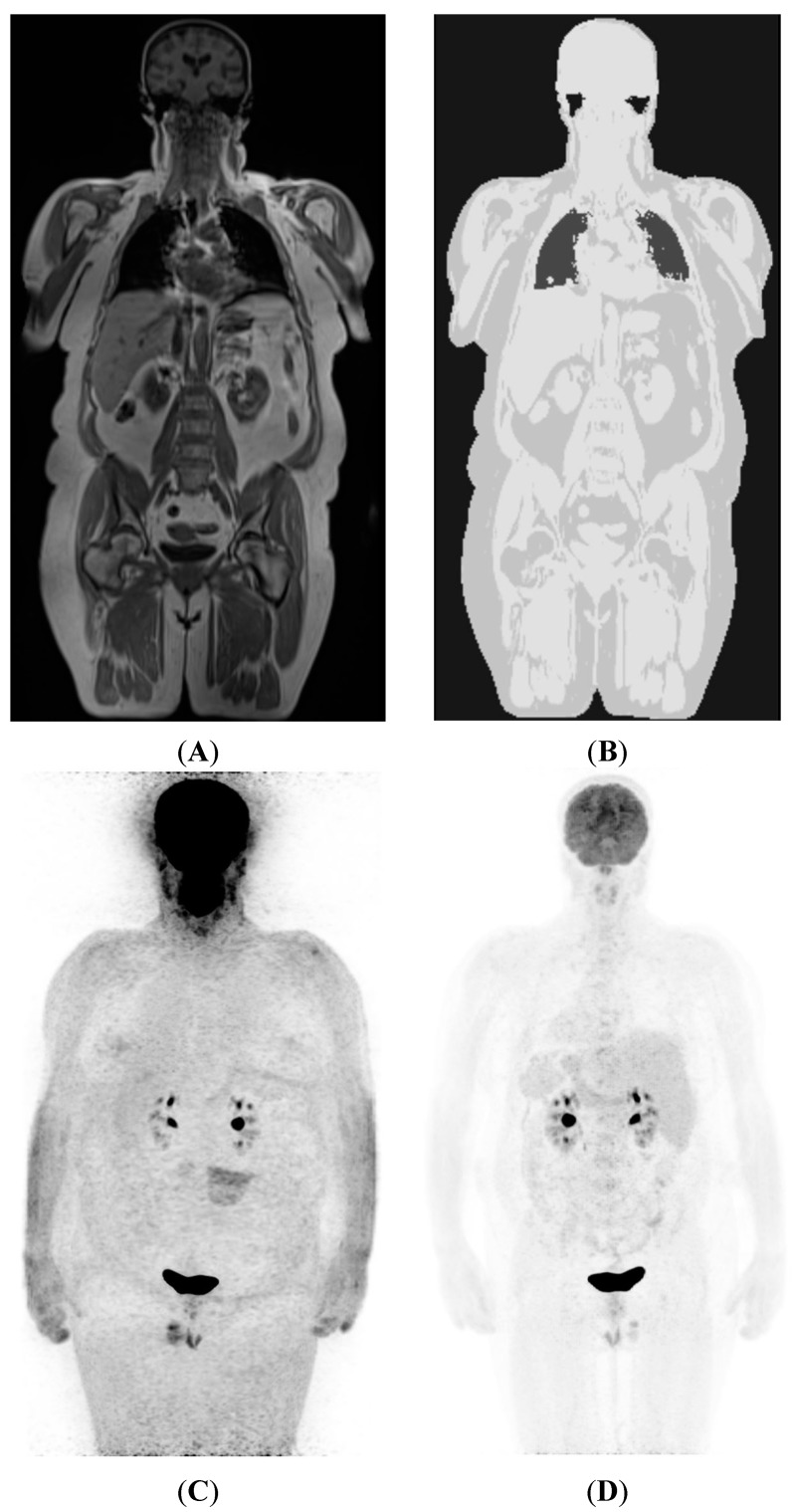
(**A**) Coronal plane image from the Dixon VIBE sequence used to generate a MR AC map; (**B**) shows attenuation map generated from (**A**). Note four gray levels separating air, lung, fat, and soft-tissue. Also note that while bone marrow appears similar to fat elsewhere, bone cortex and overlying muscles have been assigned similar attenuation values. Reliable separation of bone in AC maps is an area of active research; (**C**) A non-corrected ^18^F-FDG PET image, showing non-uniform tracer distribution with spuriously higher uptake in peripheral tissues due to greater attenuation of emission photons from deeper structure; (**D**) AC-corrected image after applying MRI-based attenuation map shown in (**B**).

*AC-specific sequences*: These sequences are usually acquired before diagnostic sequences. 2-point Dixon volume-interpolated breath-hold examination (VIBE) sequences are fast and allow derivation of an attenuation map based on four tissue-types: air, lungs, soft-tissue, and fat. However, due to the signal void within cortical bone, bone attenuation is also categorized as soft-tissue and this may lead to underestimations of SUVs in bone lesions by as much as 30% [3,14,15] (Figure 1). Despite the imperfect AC in the current MRI-based AC techniques, several *in vivo* studies on patients have not shown a significant disadvantage in detection of lesions [16,17]. Nevertheless, alternative approaches utilizing ultra-short TE MR sequences may allow improved profiling of bone attenuation, although such approaches have their own limitations—artifacts at larger FOV and extra acquisition time [18].

## 3. Imaging Protocols and Workflow

Sequential PET/MRI involves longer scan acquisition times compared to simultaneous PET/MRI since the patient is imaged twice, once with PET, and once with MRI. Here, we present an overview of simultaneous PET/MRI workflow based on our experience.

Patient preparation for PET/MRI accounts for the contraindications and precautions for both modalities. Hence, as for ^18^F-fluorodeoxyglucose (^18^F-FDG) PET in PET/CT, the requirements include a four-hour fast, control of blood glucose level, and having the patient rest after FDG injection, to minimize muscle uptake. The contraindications to PET/MRI are the same as for MRI, e.g., checks for metallic implants and pacemakers, and for PET, e.g., pregnancy.

Patient positioning for PET/MRI needs to be more precise than is necessary for PET, especially with regards to patient centering on the imaging couch and positioning of the surface coils, to optimize the MRI-image signal and avoid artifacts. Hence, positioning takes longer than it does for routine PET, and can potentially add to staff radiation. Therefore, staff should preferentially be trained in both PET and MRI.

**Figure 2:**
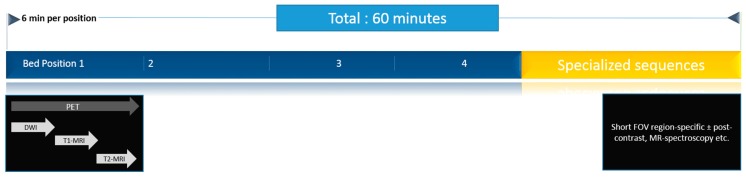
A typical PET/MRI workflow, as performed in our institution. Patients are scanned in four bed positions to cover the anatomy from head to mid-thighs. Whole body imaging is performed first, followed by targeted sequences for the region of interest. Typically, a scan lasts for 60 min.

Once the patient has been positioned, an MRI localizer sequence (analogous to scout scan in CT) is acquired to determine scan range. Thereafter, PET acquisition is straightforward, requiring input of the number of bed positions and time at each bed position (typically 3–4 min but can be extended to make use of the longer MRI scan-time). Concurrent to PET acquisition, an MRI attenuation correction sequence (2-point Dixon VIBE) is acquired, followed by the diagnostic MRI sequences for the current bed position. A number of diagnostic MRI sequences may be selected and can be varied flexibly according to body region and clinical question, but usually at a minimum include a T1-weighted and a T2-weighted sequence[19]. A typical PET/MRI workflow is illustrated in Figure 2.

A “minimalist” PET/MRI protocol including only 2-point Dixon VIBE for both AC and anatomic localization has been validated and takes less than 20 min [17]. On the other hand, a typical whole body study including organ-targeted sequences can easily take 60-min or more, compared with about 15–30 min typical for PET/CT. The longer time required to perform PET/MRI is an important issue and will result in costlier studies compared with PET/CT, which must be borne in mind while justifying its financial feasibility in clinical settings [19,20].

## 4. Overview of Cancer Imaging

PET/MRI is expected to supersede PET/CT in imaging cancers which are anatomically better defined by MRI compared to CT, due to its superior soft-tissue contrast. These include brain, head and neck, breast, liver, musculoskeletal system, and urogenital tumors [21]. New applications, not addressed by CT, may also become apparent. For example, MRI sequences for the measurement of tumor perfusion (*i.e.*, dynamic contrast-enhanced MRI or arterial spin labelling) and hypoxia (e.g., blood-oxygen-level-dependent “BOLD-MRI”) may be used independently or to cross-validate PET tracers for similar purposes, such as ^15^O-H_2_O (perfusion) or ^18^F-fluoromisonidazole (hypoxia). Complementary data from PET tracers and MRI sequences designed to answer similar questions may provide additional information. Given below is an overview of the current literature on applications of PET/MRI divided into three sections: organ-based assessment of local tumor, detection of lymph node metastases, and detection of distant metastases.

## 5. Assessment of Local Tumor

The majority of early studies assessing feasibility and accuracy of PET/MRI in oncology are on pelvic, breast, brain, and head and neck malignancies. Since retrospective fusion of images acquired on separate PET and MR scanners has been available for longer, there are more studies based on image-fusion than on hybrid (simultaneous or sequential) PET/MR imaging. Although retrospective software-based PET and MRI fusion allows a degree of flexibility in choice of co-registration algorithm on a case-to-case basis, hybrid PET/MRI acquisition generally offers superior co-registration because the patient remains in the same position for both modalities [22,23].

### 5.1. Head and Neck

^18^F-FDG the most commonly used tracer in whole body PET/CT imaging, has low sensitivity to brain lesions due to high background activity[24]. Likewise, contrast-CT is limited in delineating small lesions, compared with MRI [25]. Hence, PET/MRI may prove superior to PET/CT in brain-tumor imaging, especially sub-centimeter metastases which profoundly influence treatment decisions (*i.e.*, metastasectomy, whole brain radiation, or gamma-knife surgery). Studies have shown that PET/MRI with brain tumor-targeted tracers such as [^11^C]methionine and ^18^F-fluoro-ethyl-tyrosine (^18^F-FET) is feasible, and is as accurate as PET/CT [26,27]. However, most studies describe imaging of primary brain tumors, and there is a paucity of data on brain metastases.

In staging of head and neck cancers, MRI, CT, and PET/CT have overlapping advantages. Most studies have shown CT to be superior to MRI in detecting bony cortex invasion, a marker of T4 stage [28,29,30,31]. By comparison, MRI is more reliable in detecting bone marrow invasion, invasion of deep tongue muscles, perineural spread, and intra-cranial extension [28]. ^18^F-FDG PET/CT, on the other hand, outperforms both MRI and CT in detecting metastatic lymph nodes, occult primaries, and distant metastases. Despite its advantages, PET/CT is not recommended in primary staging of neck-negative (N0) cancers due to low pre-test probability of nodal or distant metastasis, and MRI or CT is considered sufficient in this scenario [32].

**Figure 3:**
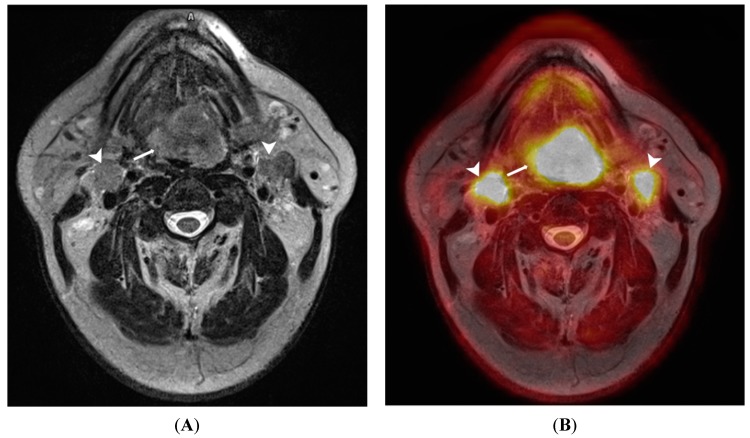
A 65-year old male patient with lingual carcinoma (**A**) T2-weighted MRI and (**B**) ^18^F-FDG PET/MRI. The primary tumour (arrows) and nodal metastases (arrowhead) are considerably more conspicuous on PET/MRI compared with T2-weighted images. This advantage could be exploited in detecting small occult head and neck tumours.

Compared with conventional imaging modalities, ^18^F-FDG PET/MRI has been shown to be feasible in head and neck cancer staging [33,34] (Figure 3). Indeed, there is evidence that PET/MRI may be better at local tumour assessment compared with PET/CT, CT, or MRI. A recent study compared FDG-PET/MRI with PET/CT over 149 lesions in head and neck patients referred for baseline staging (30%) or follow-up (70%). For PET/MRI, the authors reported an overall higher diagnostic confidence in characterizing local tumour, whereas both modalities were comparable in detecting metastastic lymph nodes. Not surprisingly, PET/MRI with contrast-enhanced sequences, was better at detecting perineural spread (*n* = 3), and PET/CT was superior in assessing larynx and bone cortex (2 patients). Huang *et al.* [35] compared T-staging accuracy of software-fusion PET/MRI, PET/CT, MRI, and CT in 17 patients with buccal carcinoma. Histopathology was the reference standard.The authors found that PET/MRI had the highest positive likelihood ratio (43) compared with PET/CT (25), MRI (25), and CT (9), proving superior to the other modalities in accuracy and positive and negative predictive values. They also found that PET/MRI measured maximal tumour diameter had the highest degree of correlation (*r*^2^ = 0.96) with histopathology. They concluded that fused PET/MRI is more reliable for focal tumour invasion and size assessment than PET/CT, CT, and MRI.

Contrary to above findings, Nakamoto *et al.* [36] did not observe a benefit of PET/MRI compared with MRI alone in initial staging of patients with head and neck cancer. In their experience, software fusion PET/MRI only detected one additional tumour in patients undergoing initial staging (*n* = 46). In contrast, fusion PET/MRI was considerably better than MRI in patients with suspected recurrence (*n* = 15; sensitivity 92% *vs.* 67% for MRI alone).

It should be noted, however, that most studies to date have included small heterogeneous patient groups, and have used variable MRI sequences and parameters. We believe that well designed PET/MRI studies on larger populations will identify a role for PET/MRI in scenarios which would benefit from combined metabolic and high-contrast anatomic imaging. These will include patients with suspected recurrence in whom local anatomy has been disturbed with treatment, patients with occult small primary tumours, and patients with tumours close to skull base where CT is limited compared with MRI.

### 5.2. Lung Cancer

The current imaging modalities offer different advantages in lung tumor evaluation: Contrast CT, due to its high spatial resolution, is accurate in T-staging of lung cancer [37], and is the most commonly used modality in initial work-up. However, it does not delineate post-obstructive collapse from tumor, thereby causing inaccurate size-estimation, which can affect management decisions (for example, determining T stage and choice of radiation field). ^18^F-FDG PET does not suffer from this short-coming because it relies on the increased metabolism of the tumor to differentiate it from adjacent lung collapse. MRI, however, is best suited for superior sulcus tumors and tumors close to the mediastinum, because it can differentiate tumor from brachial plexus and mediastinal fat, respectively [36,38]. Combined PET and MRI may prove useful in initial staging of suspected superior sulcus tumors and tumors abutting the mediastinum with MRI providing local information for T-staging, and PET allowing M-staging. There may also be a role for better tumor profiling and phenotyping by using multiparametric functional and molecular data that may become more relevant with increasing use of personalized and targeted biological therapy.

Studies of hybrid PET/MRI have shown comparable accuracy to PET/CT. For example, Schwenzer *et al.* [39] compared PET/MRI with ^18^F-FDG PET/CT. Both modalities agreed in TNM staging in seven of 10 cases. Of the three discordant cases, none had impact on management. However, it is noteworthy that PET/MRI identified mediastinal invasion in one case, not reliably seen on PET/CT. In a similar comparative setup on 22 patients, Heusch *et al.* [40] added further chest-specific MRI sequences optimized to assess any added benefit, *i.e.*, post-contrast T1WI and DWI. The authors found 100% accuracy for T-staging for both modalities, and 91% and 82% accuracy for N-staging, for PET/MRI and PET/CT respectively. They concluded that ^18^F-FDG PET/MRI imaging with a dedicated pulmonary protocol did not confer any additional advantage over standard whole-body sequences.

Most studies using retrospective fusion PET/MRI have also shown comparable accuracy with PET/CT [39,40,41,42,43]. For example, a recent randomized controlled trial compared fusion PET/MRI (*n* = 140) with ^18^F-FDG PET/CT (*n* = 123) in patients with resectable NSCLC [43]. Both PET/MRI and PET/CT correctly upstaged 26% and 22% of the patients, respectively, showing a slight advantage for PET/MRI, although the difference was not statistically significant. Indeed PET/MRI incorrectly over-staged more patients (18% *vs.* 6%), which may cause unnecessary further investigations in practice. However, as the study was performed on 1.5T MRI and most current hybrid PET/MRI scanners are 3.0T, these results may not be generalizable to modern hybrid scanners.

### 5.3. Genitourinary System Malignancy

Management decisions for prostate cancer mainly depend upon TNM stage, tumor grade, and PSA level. Local staging of prostate cancer is usually performed with multi-parametric MRI (T2-weighted MRI and a combination of DCE, DWI, or MR spectroscopy sequences) [44]. It has a sensitivity and specificity of 74% and 88% in detecting prostate cancer [45], and 86% and 95% in determining extra-capsular extension (ECE) [46]—a marker of locally advanced disease necessitating aggressive management. On the other hand, PET with newer tracers such as ^11^C or ^18^F-choline, ^18^F-FACBC or ^68^Ga-PSMA shows promise in detecting distant metastasis, and in diagnosing local relapse. Tumor grade is usually determined with trans-rectal or trans-perineal biopsy, although it is prone to sampling error [47]. Values derived from PET and MRI also correlate with Gleason score, but considerable overlap limits their usefulness [48,49,50]. By combining these features, PET/MRI is expected to improve tumor grading and aid in the detection of relapse. It may also provide a one-stop staging for high-risk patients, who would otherwise require a separate bone scan for skeletal metastases and/or CT scan for retroperitoneal nodes.

**Figure 4:**
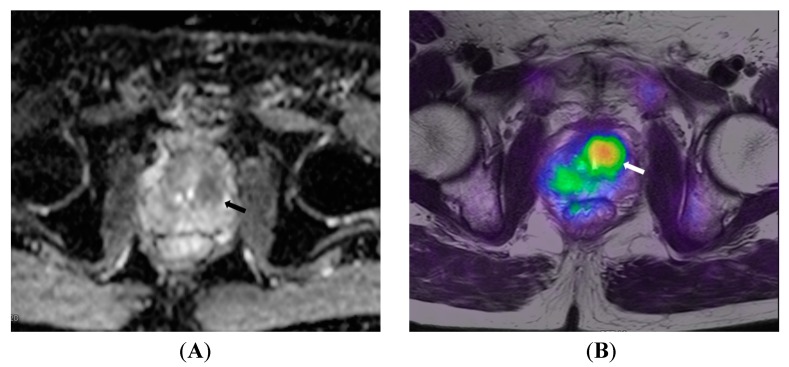
A 72-year old patient with prostate cancer. (**A**) ADC map shows low signal intensity in the central gland (arrow), which is confirmed by 18F-choline PET/MRI (arrow in **B**) to be metabolically active central gland tumor.

Early studies on PET/MRI suggest that it may be as accurate as or superior to ^18^F-choline PET/CT in detecting prostate cancer [51,52,53,54]. A study of 32 patients found that both modalities concurred on 77 of 80 primary prostate lesions, with PET/CT missing one prostate lesion and PET/MRI missing one bone and three nodal lesions. Additionally, PET/MRI showed better anatomic localization of active lesions than PET/CT. In another series of 23 patients, ^18^F-fluorocholine PET/MRI performance was similar to multiparametric MRI in detecting prostate cancer[52]. However, multivariate analysis on the subset of peripheral zone tumors showed higher detection accuracy for PET/MRI parameters (ADC + SUV_max_) than multiparametric MRI (ADC + T2 + extravascular extracellular volume fraction), with an AUC of 0.89 *vs.* 0.84. Central zone tumors, on the other hand, were difficult to evaluate with both modalities, due to coexisting benign prostatic hyperplasia. This peripheral/central zone differentiation is not possible with PET/CT. Figure 4 illustrates the utility of PET/MRI in detecting central gland tumors.

In tumor grading, there is evidence that combined PET/MRI may improve the accuracy of either modality alone [55,56]. Park *et al.* [55] tested the combined parameter from fusion ^11^C-choline PET/MRI data, *i.e.*, P_CHOL/ADC_ ratio, in 17 patients. Following imaging, all patients underwent radical prostatectomy. To identify tumors on the fused images, the authors co-registered the images with histologic sections. They found that the P_CHOL/ADC_ ratio from fused PET/MRI correlated with tumor grade better than either PET or MRI. The authors observed some overlap; P_CHOL/ADC_ ratio values did overlap between low-grade and high-grade tumors, albeit less than with PET or MRI alone. They suggested this overlap may be due to unavoidable registration with software fusion, and may be further improved with hybrid PET/MRI scanners.

The above studies show that ^11^C or ^18^F-choline PET/MRI is a possible superior alternative to PET/CT in prostate cancer imaging, and perhaps multiparametric MRI too. Although it may be hard to justify PET/MRI in baseline staging of most patients, it may be useful in staging advanced prostate cancer with suspected bone metastasis and in detecting relapse in patients with rising PSA levels following treatment [57].

### 5.4. Gynecological Malignancy

MRI is the dominant local staging tool for endometrial and cervical cancers. For ovarian cancer, CT is preferred for staging due to its wide availability and equivalent performance to MRI. PET/CT with its whole body coverage is justified in high risk patients for detecting distant metastases and in suspected relapse. However, due to the poor accuracy of current imaging tools in lymph node staging and detection of peritoneal carcinomatosis, surgical staging remains necessary [58,59].

Despite the role of MRI in staging uterine malignancy, and PET/CT in ovarian cancer, to date only a few studies have been performed on combined 18F-FDG PET/MRI. One study on biopsy proven endometrial cancer (*n* = 30) found that PET/MRI detected more lesions than PET/CT (97% *vs.* 93%) and was more accurate in T-staging (80% *vs.* 60%). For N-staging both modalities were comparable and superior to MRI (100% *vs.* 67%). These findings agree with the known strengths of MRI and PET in local tumor and nodal staging respectively. With regards to detection of recurrence of gynecological malignancies, there is evidence from small studies (*n* < 20) that both PET/MRI and PET/CT are equally effective [60,61]. It will be interesting to know how PET/MRI performs in staging ovarian cancer, especially in detection of peritoneal implants where DW-MRI has shown promise [62].

### 5.5. Gastrointestinal Tract Cancers

Characterization of hepatobiliary system (HPB) tumors can be challenging due to the myriad of overlapping appearances shared among the various benign and malignant pathologies. Although PET is more sensitive than CT in detection of HPB tumors, it does not have a role in local evaluation except in cases of recurrence or unknown primary. Similarly, the assessment of luminal gastrointestinal tract is best performed by MRI and endoscopic ultrasound, due to their superior resolution in differentiating mural layers.

There are a few studies evaluating the performance of PET/MRI in GI malignancies. One study assessing rectal cancer staging with fusion ^18^F-FDG PET/MRI (*n* = 23) did not find it superior to MRI plus abdominal CT and chest radiography. According to another study on staging of esophageal cancer [63], although PET/MRI was superior to PET/CT in local staging, it was inferior to EUS. The results from these studies have not shown that PET/MRI is of added benefit to patients undergoing routine staging in GI cancers, but there are a number of further unexplored GI applications that may be relevant.

### 5.6. Breast Cancer

MRI is believed to be the most sensitive modality to detect primary breast cancer with reported sensitivities of 89%–100% [64,65,66,67,68,69,70,71,72,73,74,75,76]. However, as shown in a multi-center trial on breast cancer screening, it has a limited positive predictive value of 43% [77]. Therefore, it is only recommended in specific populations, such as screening patients at high-risk (e.g., due to genetic predisposition or previous mantle radiotherapy for lymphoma), to determine the extent of lobular carcinoma or ductal carcinoma-in-situ (frequently occult on other imaging), and in neoadjuvant response assessment. Likewise, FDG PET/CT is not recommended for routine screening or workup for breast cancer due to its propensity to miss sub-centimeter or low-grade cancers [78]. However, it can be used to stage locally advanced cancers with high pre-test probability of axillary metastasis; in such patients it can obviate sentinel node biopsy [79,80,81,82]. Several authors have studied the added advantage of ^18^F-FDG PET to breast MRI. According to one report, fusion PET/MRI lowered the number of false positive cases on MRI from 16 to 1[83]. Although fusion PET/MRI also missed more cancers than MRI alone (8 *vs.* 2), the latter finding was not statistically significant. In another study, hybrid PET/MRI detected all 10 sub-centimeter IDCs, of which only 1 showed FDG uptake on PET [84]. These findings suggest two advantages for PET/MRI. First, in patients undergoing routine staging, it seems to overcome the shortcoming of PET in detecting small and well-differentiated carcinomas, thus allowing a more comprehensive one-stop staging modality. Second, it seems superior to breast MRI due to higher specificity whilst allowing the dynamic MRI sequences. However, it is unlikely to be used in screening high-risk patients like MRI, due to radiation risks.

## 6. Nodal Assessment

Detecting metastatic lymph nodes is an essential part of staging work-up. However, imaging has historically been limited in nodal staging, and is supplemented by other invasive and non-invasive techniques. CT and MRI diagnose nodal metastasis based on size, nodal contour, and appearance [85,86,87,88]. In comparison, ^18^F-FDG PET is more sensitive, with a reported sensitivity of 61%–87%, and specificity of 84%–99% [89,90,91]; hence it is frequently used when CT or MRI are equivocal. All imaging modalities frequently under report sub-centimeter lymph node metastases [92,93,94,95,96,97].

To date, most studies evaluating nodal staging with PET/MRI are heterogeneous in patient populations, choice of MRI sequences, and reference standards. They report mixed results regarding the accuracy of PET/MRI in detecting metastatic lymph nodes. For example, Platzek *et al.* [98] assessed 391 lymph node stations in 38 patients with untreated head and neck cancer before surgical neck dissection with sequential PET/MRI. They found that PET/MRI did not add any advantage to PET alone (sensitivity and specificity of 90% and 95% respectively *vs.* 87% and 97%). Similar results have been shown in other populations of head and neck and chest malignancies [99,100]. In contrast, Kim *et al.* [101] fused PET and MRI images in 79 patients with cervical cancer undergoing baseline work-up. With the help of fusion images, they detected nodal metastases in three additional patients, node-negative on PET/CT. They concluded that fusion PET/MRI improved the detection of lymph node metastases in patients with cervical cancer.

**Figure 5:**
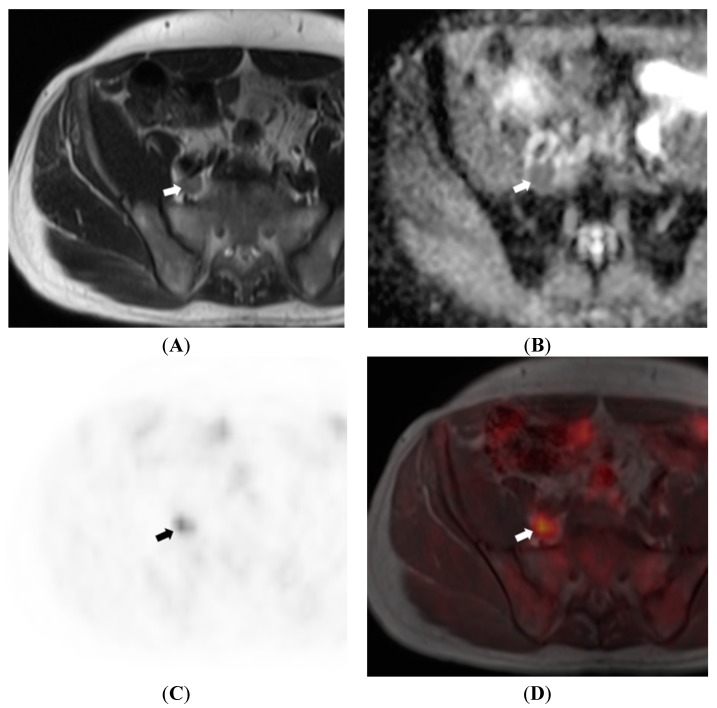
64 year old male patient with prostate cancer. T2WI (**A**) show a large right common iliac lymph node (arrow) which exhibits restricted diffusion on ADC map (**B**); ^18^F-choline PET (**C**) and fusion PET/MRI (**D**) confirm the lymph node to be metabolically active, further increasing confidence regarding it being metastatic.

It is unknown whether MRI, with its superior soft-tissue contrast, can improve the combined accuracy of a PET/MRI scanner as compared to PET/CT in detecting malignant lymph nodes. As the individual components of PET/MRI are only modestly accurate, and limited in detecting micro-metastases, there is a need for research into newer PET tracers, MRI contrast agents such as USPIO, and MRI techniques. Studies assessing the additive value of DWI sequences with PET also need to be performed because DWI sequences have been shown to be more accurate than standard MRI sequences [102] (Figure 5). Indeed, ongoing animal studies employing nanoparticles as PET tracers are showing promising results [103,104].

## 7. Metastases

### 7.1. Liver

Multi-parametric MRI performed with liver-specific contrast agents allows confident differentiation of various types of benign and malignant entities, which may appear indeterminate on CT or ultrasound. Furthermore, it can detect sub-centimeter lesions below the resolution of PET or CT, which influence management [105,106,107,108]. Therefore, whole body applications of PET/MRI incorporating liver-specific MRI sequences are expected to outperform PET/CT in screening for liver metastasis.

Studies of both fusion and prospective hybrid PET/MRI show promising results. Beiderwellen *et al.* [109] performed simultaneous PET/MRI following ^18^F-FDG PET/CT in 70 patients with various cancers. They used iodinated contrast for CT, and for MRI, they used whole-body imaging without specialized liver sequences. They found that both modalities performed equally well and detected all metastatic lesions (*n* = 26), whereas PET/MRI allowed more accurate characterization of benign lesions (*n* = 71). On the other hand, Donati *et al.* detected significantly more metastases with software-fusion ^18^F-FDG PET/MRI than with PET/CT (93% *vs.* 76%) [105]. In their study on 37 patients, they included dynamic MRI sequences after injection of liver-specific contrast agents (gadoxetic acid) retrospectively fused with PET images acquired as part of an ^18^F-FDG PET/CT study. The authors, concluded that both gadoxetic-acid enhanced MRI alone and software-fusion PET/MRI were more sensitive than PET/CT in detecting liver metastasis. Other researchers evaluating PET/MRI in neuroendocrine tumors drew similar conclusions [110].

### 7.2. Bone

Of the conventional tools evaluating bone metastases, bone scintigraphy or ^18^F-FDG PET are considered to be the most sensitive in ^18^F-FDG-avid malignancies. CT has low sensitivity to marrow lesions before the appearance of reactive bone sclerosis or cortical destruction. MRI is superior to CT but takes much longer in whole body acquisition; therefore, it is usually performed as a second-line tool for trouble-shooting or for assessment of complications (e.g., in the spine). Whole-body MRI protocols, especially with diffusion-weighted imaging, are promising in bone-metastasis screening, but are not yet in widespread use for this purpose. These different strengths of PET and MRI can be exploited in a one-stop solution for bone metastasis assessment, with sequences tailored to individual indications.

Several studies have assessed various PET/MRI sequences and have shown them to be comparable to PET/CT in performance. For example, Eiber *et al.* [111] found that whole body T1-weighted sequences in simultaneous FDG-PET/MRI detected a similar number of bone metastases to PET/CT (84 *vs.* 86 out of 90). Moreover, they reported superior anatomic correlation for T1-weighted MRI sequences compared with CT. Beiderwellen *et al.* [112], conversely, found that PET/MRI with DWI and post-contrast T1-weighted imaging detected a slightly greater number of malignant lesions than PET/CT (48 *vs.* 45 out of 48). However, PET/CT detected more benign lesions than PET/MRI (26 *vs.* 18 out of 27), all of which were sclerotic. Both groups concluded that PET/MRI is a viable alternative to PET/CT for bone metastasis detection and allows improved lesion conspicuity and anatomic localization.

## 8. Ongoing Challenges and Future Directions

For PET/MRI to gain a place among modern cancer imaging, several issues must be addressed. New clinical research should be of different design and larger scale. This is because most studies to date are on small patient populations and have addressed generic questions, such as baseline staging and recurrence in heterogeneous populations. These studies have not shown an overwhelming advantage of PET/MRI compared with PET/CT. For future studies, a paradigm shift is needed to answer more specific questions, and to identify new clinical indications. For instance, rather than direct comparison with PET/CT, the efficacy of PET/MRI may be tested in MRI-specific scenarios [113]. Secondly, since MRI scan times are the limiting factor, there is a need for studies testing the optimal MRI sequences to combine with PET imaging to give the most useful complementary information. These may include functional imaging sequences such as DWI, DCE-MRI, ASL and BOLD MRI, and different PET tracers such as ^18^F-FLT and ^18^F-fluoromisonidazole. In this respect, this is evidence that the addition of DWI to standard whole-body sequences may not be advantageous in evaluating tumors involving the lungs [40] or head and neck [114]. Thirdly, logistics, time and work-flow, and cost considerations need to be addressed because hybrid PET/MRI scanners are expensive and examinations typically last longer than PET/CT studies. Lastly, there are outstanding technological challenges which must also be addressed. For example, AC based on MRI is still imperfect, and is a subject of active research [13].

## 9. Conclusions

PET/MRI is a promising new technology with early studies suggesting that it may have a role in most aspects of oncological imaging from diagnosis to response assessment and surveillance. Although some studies have not shown a clear advantage over PET/CT, there is some evidence that it will have an advantage in selected body sites such as the head and neck, liver, and the pelvis. Another benefit will be logistical by allowing selected patients to undergo a comprehensive one-stop examination rather than separate studies. However, clinical deployment will still need to be balanced against the cost of a PET/MRI scanner. With further maturity of PET/MRI equipment, and with larger studies in different scenarios, we expect clearer answers to most of these questions.
